# The Use of Survival Dose-Rate Dependencies as Theoretical Discrimination Criteria for *In-Silico* Dynamic Radiobiological Models

**DOI:** 10.1177/15593258241279906

**Published:** 2024-08-30

**Authors:** Sergio Mingo Barba, Fernando Lobo-Cerna, Przemek M. Krawczyk, Marco Lattuada, Rudolf M. Füchslin, Alke Petri-Fink, Stephan Scheidegger

**Affiliations:** 1 School of Engineering, 30944 Zürich University of Applied Sciences (ZHAW), Winterthur, Switzerland; 2 Chemistry Department, 425660 University of Fribourg, Fribourg, Switzerland; 3 Adolphe Merkle Institute, University of Fribourg, Fribourg, Switzerland; 4 Department of Medical Biology, Amsterdam University Medical Centers, 522567 University of Amsterdam, Amsterdam, The Netherlands; 5 Cancer Center Amsterdam, Amsterdam, The Netherlands; 6 European Centre for Living Technology, Venice, Italy

**Keywords:** repair kinetics, dose-rate, radiobiological models

## Abstract

**Introduction:**

Cell repair dynamics are crucial in optimizing anti-cancer therapies. Various assays (eg, comet assay and γ-H2AX) assess post-radiation repair kinetics, but interpreting such data is challenging and model-based data analyses are required. However, ambiguities in parameter calibration remain an unsolved challenge. To address this, we propose combining survival dose-rate effects with computer simulations to gain knowledge about repair kinetics.

**Methods:**

After a literature review, theoretical discriminators based on common fractionation/dose-rate-related effects were defined to discard unrealistic model dynamics. The Multi-Hit Repair (MHR) model was calibrated with canine osteosarcoma Abrams cell line data to study the discriminators’ efficacy in scenarios with limited survival data. Additionally, survival dose-rate-dependent data from the human SiHa cervical cancer cell line were used to illustrate the survival behavior at diverse dose-rates and the capability of the MHR to model these data.

**Results:**

SiHa data confirmed the validity of the proposed discriminators. The discriminators filtered 99% of parameter sets, improving the calibration of Abrams cells data. Furthermore, results from both cell lines may hint universal aspects of cellular repair.

**Conclusions:**

Dose-rate theoretical discrimination criteria are an effective method to understand repair kinetics and improve radiobiological model calibration. Moreover, this methodology may be used to analyze diverse biological data using dynamic models *in-silico*.

## Introduction

**Figure 1.**
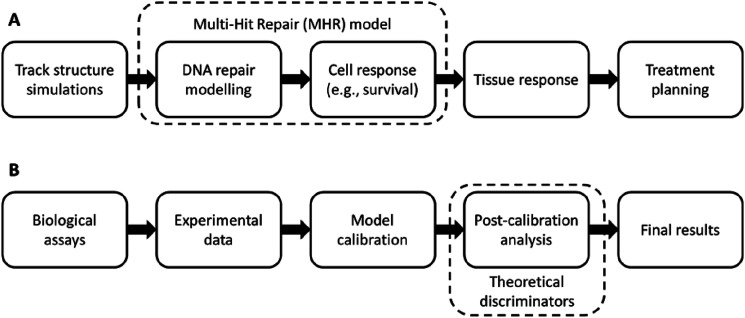
Schematic overview of radiobiological models and their calibration/validation process. (A) Illustration of the radiation effects at different scales, which can be captured by *in-silico* models and the coverage of the MHR model (dotted box). (B) Illustration of the required steps to obtain a proper model calibration and where this work takes effect (dotted box).

Knowledge about cell damage and repair dynamics is key to developing and optimizing anti-cancer therapies. For fractionated radiotherapy (RT) or the combination of hyperthermia with radiation (HT-RT), the knowledge about repair kinetics would support therapy optimizations such as the adaption of fractionation or, in the case of HT-RT, the selection of appropriate time gaps (time intervals) between heating and irradiation. The time gap dependence of the synergistic effect between radiation and heat after HT-RT can be assumed to be influenced by the repair speed of radiation-induced damage and the kinetics of repair protein inhibition.^1^ To investigate and understand this aspect, fitting *in-silico* dynamic models to experimental data *in-vitro* and *in-vivo* may support a deeper insight into the dynamic processes governing the therapy outcome. However, a problem arising with the multi-scale structure of biological systems is the link of processes taking place at lower scales (repair of DNA double-strand breaks or initiation of apoptotic signaling pathways) to the dynamic response on higher levels (emergent cell reaction, tissue dynamics including tumor-host interaction, competition or immune system activation). A description of these upper-scale processes would require a model for cell population interaction (ecosystem model). This leads to the question of how DNA repair processes can be included in a dynamic model *in-silico* based on repopulation/reproduction and elimination of cells. This was one of the main motivations for the *in-silico* model used in this manuscript, the Multi-Hit-Repair (MHR) model.^1^ This model describes the survival on the level of the cell population to infer aspects of ecosystem and tissue dynamics (Figure 1(A)).

At the moment, different biological tests *in-vitro* can provide information about cell damage and repair dynamics. For example, the alkaline/neutral comet assays provide a relative measurement of the Single Strand Breaks (SSB) and Double Strand Breaks (DSB), respectively, produced within the cell after exposure to any stress.^2,3^ Furthermore, the amount of diverse repair proteins, such as 53BP1^4^ or Rad52,^5,6^ can be measured to investigate whether specific repair pathways are active. Also, certain assays are focused on the phosphorylation of repair proteins as γ-H2AX to obtain a time-resolved measurement of the repair kinetics.^7,8^

All the assays mentioned above seek a “direct” insight into the repair kinetics. However, cell repair involves diverse mechanisms that are usually mixed up,^9,10^ and valuable information can be missed if attention is focused on a single repair pathway. Additionally, the interpretation of the previously mentioned assays is not always clear. For example, the γ-H2AX assay has a time delay after the damage is created,^7,8,11^ the foci do not always correspond to DSB,^12,13^ and they can remain active even after the damage is fully repaired.^12-14^ Consequently, even though these assays can provide interesting dynamic information, the “direct” acquisition of accurate repair kinetics knowledge is challenging. For this reason, experiments providing more “indirect” information about the repair dynamics could sometimes be a better approach, primarily when the experiments are related to a relevant outcome, such as cell survival.

An example of a method to “indirectly” obtain information about the repair kinetics is the dose-rate survival effect. Cells exposed to ionizing radiation usually show a linear-quadratic (LQ) survival curve within the clinically relevant range of doses (<10 Gy) and dose-rates (few Gy/min) used in external beam radiotherapy.^15,16^ However, the dose-rate may affect the survival curve shape: during the irradiation, cells accumulate sub-lethal damages which can either generate lethal damages or be repaired. Therefore, if the irradiation time is increased by reducing the dose-rate, repair processes can reduce the accumulated sub-lethal damage within the cell. This increases the final cell survival by reducing the sub-lethal damages before they become lethal.^17-20^ Thus, the differences in the survival curves at diverse dose-rates are strongly related to the cell repair kinetics.

In the past, several authors have investigated dose-rate dependencies by analyzing *in-vitro* clonogenic survival,^21-29^ *in-/ex-vivo* experiments,^30-34^ or clinical trials.^35-37^ Nevertheless, these studies were mainly focused on the impact of dose-rate on clinical treatments. Ling et al.^38^ found that, for external radiotherapy, the dose-rate effect seems to be determined more by the beam-on time than by the accelerator’s average dose-rate or the instantaneous dose-rate within a radiation pulse. Additionally, for standard radiation therapy (RT) fractionations with typical dose fractions of 2 Gy, dose-rate effects may not be as important as for large doses per fraction when linear-quadratic-linear (LQL) survival shapes are expected.^39,40^

Besides its clinical importance, dose-rate dependencies can be used to understand the dynamics of the cellular response to radiation. This understanding is crucial for the development and calibration/validation of dynamic radiobiological models, which may support the optimization of RT treatments.^41^ In the literature, several examples of dose-rate/fractionation experiments are used to fit radiobiological models.^19,24,28,40^ However, dose-rate/fractionation experimental data for a specific cell line may not be accessible, making more challenging to obtain a proper model calibration. Nevertheless, specific dose-rate effects are qualitatively common to most cell lines, and they can be used to, at least, prevent the model results from being unrealistic.

In this context, our *in-silico* study aims to investigate how survival dose-rate effects can be used as a validation procedure (Figure 1(B)) to improve the calibration of *in-silico* radiobiological models (improving the understanding of the cell repair dynamics), especially when there is a lack of experimental data for a specific cell line. Therefore, this paper presents several theoretical discriminators that use the radiation response of cells to different dose-rates. As a showcase, the MHR model is selected since this model can generate survival curves^1^ as well as synthetic comets (ie, DNA damage),^42^ which can be compared to the corresponding assays *in-vitro* (Figure 1(A)). Moreover, Weyland et al.^43^ demonstrated that a combined model calibration considering survival and comet data is insufficient to determine an unambiguous set of model parameters, ie, multiple sets of parameters/repair dynamics can explain the experimental data. This leads to the conclusion that more information about the repair kinetics is required to distinguish between the different sets of parameters. Our results reveal that implementing theoretical discrimination criteria based on common qualitative dose-rate survival effects can strongly improve the calibration of dynamic radiobiological models, especially when experimental data for a particular cell line are missing. Therefore, the methodology presented here reduces the model fitting ambiguities by combining information derived from different biological assays with theoretical dose-rate aspects. This approach may be of general interest for analyzing diverse biological data using dynamic models *in-silico*.

## Methods and Materials

### The Multi-Hit Repair (MHR) Model

**Figure 2.**
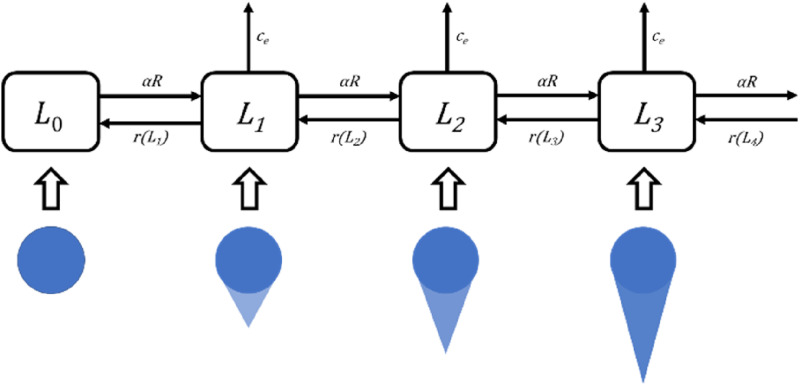
Graphical representation of the MHR model. The compartments represent the model populations *L*_*i*_ connected by arrows that symbolize how cells accumulate/repair damage (to the right/left, respectively) or undergo cell death (to the top). Under the chain, schematic comet assay figures show how the MHR model can cover biological assays measuring DNA damage: comets with higher relative tail intensities are assigned to populations with more hits (for details, see^43^). More information about the comet assay is provided in the text.

The MHR is a dynamic radiobiological model first described by Scheidegger et al.^1^ The model is divided into multiple populations *L*_*i*_, where the index *i* corresponds to the number of radiation-induced hits within a certain (critical) volume. A hit is defined as the radiation-induced damage necessary to inhibit mitotic cell duplication. By definition, only cells in population *L*_*0*_ can proliferate; therefore, their final amount will mark the efficacy of the applied treatment. The chain-like compartmental cell damage description of the MHR model is required to reproduce LQ, LQL and dose-rate survival behaviors. Also, it is one of the strongest model characteristics because, together with its dynamic nature, it allows to relate time-resolved cell damage data with the model dynamics. A graphical scheme of the model is shown in Figure 2.

In the model, hits are generated by radiation at a rate $\alpha R\left(t\right)L_{i}$ , where α is a radiosensitivity constant which is not related to the linear parameter of the LQ model^15,16^ and R(t) is the dose-rate at time *t*. For continuous irradiations, R(t) is a square pulse that starts at *t* = 0 and lasts till the irradiation finishes at *t* = *t*_*rad*_; the rest of the time R(t) = 0. The amplitude of the square-pulse, *R*_*max*_, will depend on the desired dose-rate to be applied. Hence, the irradiation time of a dose *D* is *t*_*rad*_ = *D/R*_*max*_. During the irradiation, cells are allowed to develop through the hit chain till a population with *k*_*max*_ hits. Herein, the same criterion (*k*_*max*_ = 9) followed by Weyland et al.^43^ is used. This criterion ensures proper convergence of the simulation results, as Appendix A of the Supplementary Material demonstrates. On the other hand, cells can travel in the opposite direction by repairing the radiation-induced damage. The repair is proportional to a repair rate constant $c_{r}$ , and it is modulated by a repair probability function $P_{r}\left(t\right)$ , which will be further explained later. Finally, spontaneous cell elimination is also included in the model to simulate failures in the repair leading to cell death. This cell elimination is assumed to occur at a rate $c_{e}L_{i}$ . The rate equation which governs the evolution of a population *L*_*i*_ is the following:

(1) $$\frac{dL_{i}}{dt}=\alpha R\left(t\right)L_{i-1}-\alpha R\left(t\right)L_{i}-c_{r}P_{r}\left(t\right)L_{i}+c_{r}P_{r}\left(t\right)L_{i+1}-c_{e}L_{i}$$

The model assumes that ionizing radiation does not only damage DNA, but it also can damage/deactivate repair proteins or saturate repair pathways. Reducing the available proteins within the cell during and after irradiation will decrease the cell repair probability and, consequently, slow down the repair speed. Therefore, to mimic this scenario, a Transient Biological Dose Equivalent (TBDE) Γ(t) model is used and included in the repair probability function $P_{r}\left(t\right)$ :

(2) $$\frac{d\Gamma \left(t\right)}{dt}=R\left(t\right)-\gamma \Gamma \left(t\right)$$

where R(t) is the dose-rate square pulse as explained previously, and γ is the TBDE repair rate constant. This TBDE is included in the repair probability as

(3) $$P_{r}\left(t\right)=e^{-\mu_{\Gamma }\Gamma \left(t\right)}$$

where the constant $\mu_{\Gamma }$ modulates the effect produced by the radiation-induced inactivation of repair proteins.

### Simulation Conditions

All the simulations are set to reproduce 10 hours after irradiation and the same initial conditions as the ones assumed by Weyland et al.^43^ are applied: All the cells are clonogenic (ie, *L*_*0*_ *= 1* and *L*_*i*_ *= 0* for *i* > 0 as the total amount of cells is normalized) and no repair proteins are deactivated ( Γ(0) *= 0*).

### Experimental Data

In this work, the canine osteosarcoma Abrams cell line experimental data presented by Weyland et al.^43^ were used to study the effectiveness of the presented theoretical discriminators. They measured the clonogenic survival after 3 and 6 Gy irradiations. The clonogenic assay measures the ability of cells to duplicate and form colonies (consisting of at least 50 cells) after exposure to a specific stress.^44^ Also, they measured the alkaline comet assay tail intensities at 15, 30, 60, 120, 240 and 360 minutes after a 6 Gy irradiation. The comet assay consists of single-cell gel electrophoresis for measuring DNA breaks.^3^ The damaged DNA loops can move in the electric field generating a tail. Therefore, the intensity of the comet tail relative to the head is related to the amount of DNA damage. A dose-rate of 6 Gy/min was used in all the experiments.

Additionally, the SiHa cervical cancer cell line was used to illustrate the survival behavior at diverse dose-rates and the capability of the MHR to model these data. Cell lines were cultured at 37°C in humidified air supplemented with 5% CO_2_ and maintained in EMEM (Gibco) supplemented with 10% fetal bovine serum (Gibco), 1% Penicillin-Streptomycin (Gibco) and L-glutamine (Gibco). Cells were trypsinized, counted and subcultured into flat-bottom 6-well plates (Greiner) 4 hours before irradiation. Irradiations were performed using CellRad from Precision X-ray (Madison,CT, USA) at 3 dose-rates (0.5, 2 and 6.5 Gy/min) and 5 doses (0, 2, 4, 6 and 8 Gy). Following the treatment, cells were cultured until colonies reached sufficient size (>50 cells per colony), then fixed and stained in a solution of PBS containing 0.05% crystal violet (Sigma, Saint Louis, MO, USA) and 1% glutaraldehyde (Sigma) and counted.

### Model Mapping and Comparison with Experimental Data

As the MHR model does not directly describe biological assay readouts, a mapping method is required to compare such readouts with corresponding quantities from the model. As previously mentioned, only cells in the *L*_*0*_ population have a proliferative capacity, ie, the survival fraction will equal the final value of *L*_*0*_. Thus, the surviving fraction can be calculated as a direct output of the MHR model and compared to biological experimental data. For the comet assay, a translation of the hit definition in the MHR model to the amount of DNA fragments in the comet tail is required (see Weyland et al.^43^). This is an intricate question because the ability of DNA damage to hinder a cell from mitosis may depend not only on the amount of physical damage but also on the location of the damage.^45-47^ A linear dependence between the comet assay tail intensity and the number of hits within a cell is assumed for simplicity. Furthermore, to explain both clonogenic and comet data, a value of 4% was considered for the largest tail intensity which is still mapped to *L*_*0*_. Hence, cells with relative comet tail intensities between 0 and 4% are assumed to be in the population *L*_*0*_, cells with relative comet tail intensities between 4 and 8% are assumed to be in the population *L*_*1*_, and so on. Finally, to compare the experimental and the model results, both of them are normalized, such as:

(4) $$\overset{k_{\max}=9}{\underset{i=0}{\sum }}L_{i}=\overset{k_{\max}=9}{\underset{i=0}{\sum }}{\hat{L}}_{i}=1$$

where $L_{i}$ is the experimental proportion of cells in the population *i* and ${\hat{L}}_{i}$ is the simulated value.

A critical point to acknowledge is that the total number of compartments (*k*_*max*_) and the considered range of comet tail intensities do not reflect the structure of the underlying biological processes. Therefore, they just have a binning intention to allow a comparison of the model results with the comet assay readouts. For more information about mapping the MHR model and its comparison with the experimental results, please read.^42,43^

### Fitting of the Model

The Approximate Bayesian Computational (ABC) method^48^ is used because of its ability to estimate model parameters probability distributions. These probability distributions allow the study of the fitting’s quality: A proper model calibration will exhibit peaked probability distributions, ie, unambiguously determined model parameters. On the other hand, spread probability distributions will imply that a broad range of model dynamics can explain the experimental data. Therefore, well-determined model parameters (ie, peaked probability distributions) are required to ensure the biological meaning of the parameters and model dynamics.

**Figure 3.**
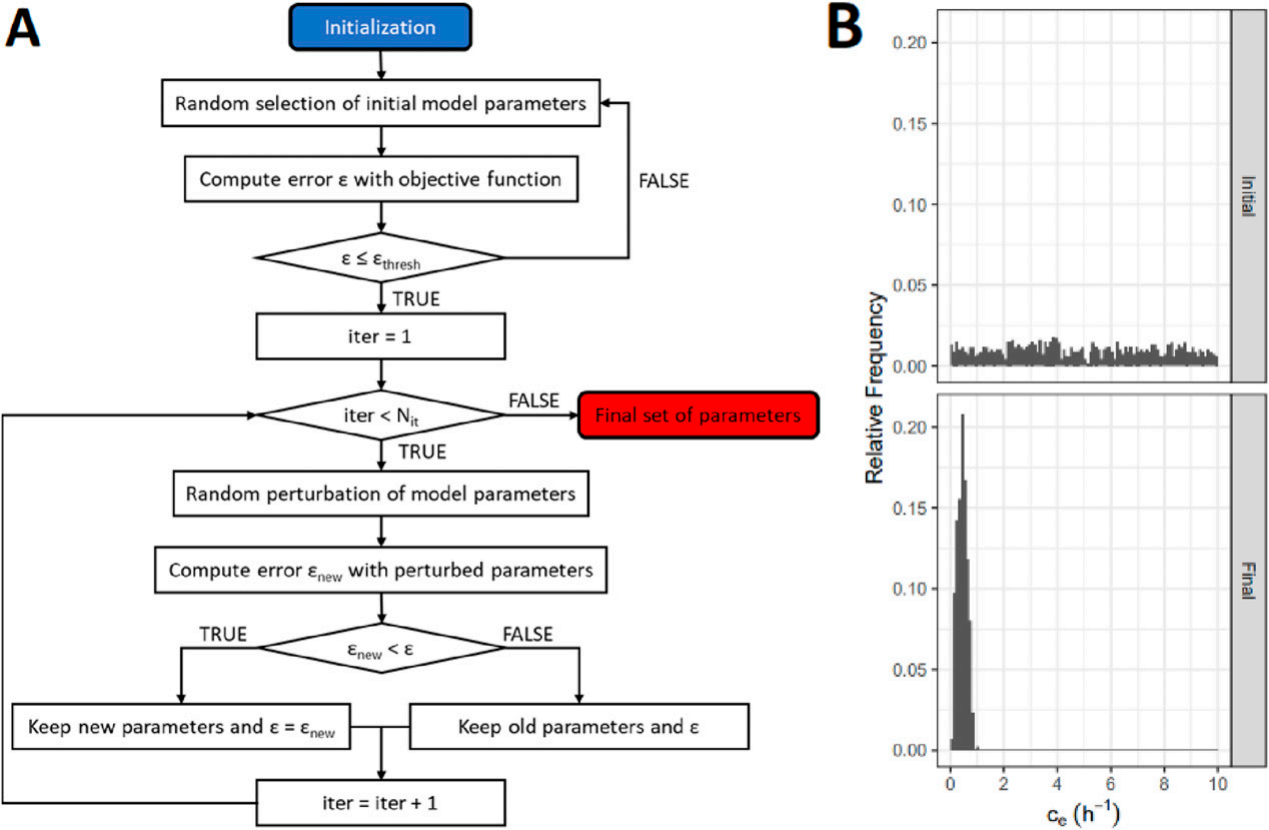
Illustration of the Approximate Bayesian Computational (ABC) calibration method workflow. (A) Process flow chart for the calibration of a single parameter set ( α *,* $c_{r}$ *,* $c_{e}$ *,* $\mu_{\Gamma }$ *,* γ ). In this work, 250 iterations (*N*_*it*_) were considered. (B) Graphical visualization of the model parameter probability distribution convergence during the calibration. In this case, the elimination rate ( $c_{e}$ ) probability distribution of 5.000 independent parameter sets (*n*) was studied at diverse stages (initial and final distributions) of the model calibration.

The ABC calibration is an iterative method (Figure 3) that works as follows: Firstly, *n* independent parameter sets (vectors with the different model parameter values) are initialized. Since there is no prior information about the parameter probability distributions, the initial parameter values are randomly selected from uniform distributions within the boundaries established in Table 1. The model is then run with these parameter values to obtain the simulated readouts, which are compared to the experimental data. The error of each parameter set is computed individually using the desired objective function (Equations (5)–(7)). In each iteration, new parameter sets are generated by adding a random Gaussian noise with a mean of zero to each parameter. The error for the new model parameters is calculated separately, and the new model parameter sets are kept if the error is reduced, and they are rejected otherwise. This process is repeated for a defined number of iterations (*N*_*it*_), after which the simulation is stopped.

**Table 1.** Summary of model parameters including their search space.

Parameter	Description	Search Range
α	Radiosensitivity (Gy^−1^)	[0.17; 2]
$c_{r}$	Repair rate constant (h^−1^)	[0; 40]
$c_{e}$	Elimination rate constant (h^−1^)	[0; 40]
$\mu_{\Gamma }$	TBDE weighting factor (Gy^−1^)	[0; 10]
γ	TBDE repair rate constant (h^−1^)	[0; 10]

The objective function to fit the comet assay data is:

(5) $$\varepsilon_{comet}=\underset{t>0}{\sum }\overset{k_{\max}=9}{\underset{i=0}{\sum }}{\left(L_{i}\left(t\right)-{\hat{L}}_{i}\left(t\right)\right)}^{2}$$

where $L_{i}\left(t\right)$ is the normalized experimental proportion of cells in the population *i* at time t and ${\hat{L}}_{i}\left(t\right)$ is the simulated value.

Similarly, the objective function for clonogenic survival data is:

(6) $$\varepsilon_{clonogenic}=\underset{D}{\sum }{\left(\log_{10}\left(S_{D}\right)-\;\log_{10}\left({\hat{S}}_{D}\right)\right)}^{2}$$

where $S_{D}$ and ${\hat{S}}_{D}$ are the experimental and simulated survival fractions at a dose D, respectively. A logarithmic objective function is employed for $\varepsilon_{clonogenic}$ rather than a linear one (as used for $\varepsilon_{comet}$ ) because the linear function underestimates the contribution of low survival (ie, high doses) experimental values.

Lastly, Weyland et al.^43^ performed separate fittings for the comet and clonogenic experimental data, and afterward, the individual parameter probability distributions were combined. In contrast, in this work, a combined objective function that uses both comet and survival experimental data is implemented (Equation (7)). Moreover, the ABC is a highly computational, time-consuming method. Therefore, it is logical to perform a single combined fitting by which the obtained results already cover both biological assays.

(7) $$\varepsilon_{combined}=\varepsilon_{clonogenic}+\xi \varepsilon_{comet}$$

where ξ is a constant used to increase or reduce the importance of one assay over the other in the final error. Here, two ξ values are used: ξ = 1, so both errors are weighted equally, and ξ = 1/30, so all the data points are weighted equally, as there are 2 survival data points and 60 comet data points.

### Dose-Rate Theoretical Discriminators

The differences observed in the survival curves at various dose-rates are highly cell line dependent. This reduces the ability to discriminate based on quantitative differences between diverse dose-rate survival curves unless real experimental values are available. However, some qualitative dose-rate effects on the survival curves, common to most cell lines, can theoretically be used to discard unrealistic repair dynamics. In such a way, it is possible to filter the sets of parameters obtained from the model fitting. This work uses different theoretical discriminators considering different aspects of the cell radiation response under diverse doses, dose-rates and fractionation schemes. These discriminators and their imposed conditions are summarized in Table 2.

**Table 2.** Summary of the defined theoretical discriminators, including the simulated doses, dose-dates, and dmposed conditions.

Discriminator	Dose-Rates (Gy/min)	Doses (Gy)	Conditions^a^
Survival at different dose-rates	0.1, 2 and 20	3, 6 and 9	(I) S(D,2)≥S(D,20) with D={3,6,9}
			(II) S(D,0.1)> S(D,2) with D={3,6,9}
			(III) log[S(6,0.1)]− log[S(6,2)]> log[S(3,0.1)]− log[S(3,2)]
			(IV) log[S(9,0.1)]− log[S(9,2)]> log[S(6,0.1)]− log[S(6,2)]
Low dose-rate limit	0.01	0 - 10	(V) Survival fitted to $\ln\left(S\right)=-\tilde{\alpha }D$ : $R^{2}\geq 0.99$ ^b^
Dose fractionation	6	6	(VI) S(3 Gy+2h+3Gy)> S(6 Gy)
Additional	0.5, 2, 6 and 6.5	0 - 30	(VII) Simulated survival at the experimental doses and dose-rates within the experimental error bars
			(VIII) Simulated survival fitted to $\ln\left(S\right)=-\tilde{\alpha }D-\tilde{\beta }D^{2}$ for *D ≤10 Gy*: $R^{2}\geq 0.99$ ^b^
			(IX) Simulated survival fitted to the LQL model presented by Guerrero et al^40^: $R^{2}\geq 0.99$ ^b^

^a^*S(D, R)* = Simulated survival at dose D (in Gy) and dose-rate R (in Gy/min).

^b^*R*^*2*^ = Coefficient of determination of the fitting.

#### Survival at Different Dose-Rates Discriminator

The survivals at 0.1, 2, and 20 Gy/min after 3, 6, and 9 Gy are simulated to study the differences in the survival that appear when cells are irradiated at different dose-rates and with different doses. At intermediate dose-rates (above 2 Gy/min), the experimental data available in the literature are quite heterogeneous, and, depending on the cell line, dose-rate effects are observed^39^ or not.^25,49^ For this reason, the condition that the survival at 2 Gy/min must be equal to or higher than the one at 20 Gy/min is imposed (condition I in Table 2). On the other hand, at lower dose-rates (between 0.01 and 2 Gy/min), dose-rate effects are more evident, and they increase with the delivered dose.^49^ Hence, two more conditions are included: the survival for a given dose at 0.1 Gy/min must be higher than the one at 2 Gy/min (condition II in Table 2), and the difference in the logarithms of the survival between both dose-rates must increase with the deposited dose (conditions III and IV in Table 2).

#### Low Dose-Rate Limit Discriminator

When the dose-rate is further reduced, the repair of sublethal damage during the irradiation starts to make the survival curve shallower until it becomes exponential.^22,30^ Therefore, the survival curve between 0 and 10 Gy is simulated at 0.01 Gy/min, and the condition that, in the logarithmic scale, the survival curve must decrease linearly with the dose is imposed (condition V in Table 2).

#### Dose Fractionation Discriminator

It is experimentally proven that fractionated dose deliveries, with fractions sufficiently separated in time, result in higher cell survival rates than acute irradiations.^29^ Hence, this discriminator simulates one acute irradiation of 6 Gy and a fractionated scheme of two fractions of 3 Gy separated by 2 hours. Then, the survivals of both irradiation schemes are compared, and it is imposed that the fractionated scheme survival must be higher (condition VI in Table 2).

#### Additional discriminators

To ensure proper coverage of the experimental data by the model, it is imposed that the simulated survival should be within the error bars of the clonogenic experimental data (condition VII in Table 2). Furthermore, it is assumed that the survival curves should have, in the logarithmic scale, an LQ shape (condition VIII in Table 2) for low/intermediate doses (<10 Gy)^15,16^ and an LQL shape for a broader range of doses (condition IX in Table 2).^39,40^

These theoretical discriminators are applied to the parameter sets obtained from the model calibration, and the parameter sets that do not fulfill these conditions are discarded.

### Statistical Analysis

At least three replicates were performed for each dose to estimate the survival fraction. The results are presented as the mean ± standard deviation.

The abcpy module^50^ of Python (version 3.9.7) is used to implement the Approximate Bayesian Computational (ABC) model calibration method previously described. In this work, the calibrations are performed with *N*_*it*_ = 250 iterations and with a cut-off value of 10^−5^. Each model calibration generates *n* = 5.000 sets of parameters. Furthermore, the numerical integration is performed with the *scipy.integrate.odeint* function with a maximum time-step of 1 s. Finally, R (version 4.1.3) is used to create the plots. All data and code used for running experiments, model fitting, and plotting are available on a GitHub repository at https://github.com/mingzhaw/Theo_discr_paper.git.

## Results

### Comparison of Experimental and Simulated Results

This work considered three objective functions (Equations (5)–(7)) to calibrate the MHR model. The model results obtained from these fittings were compared with the experimental data.

**Figure 4.**
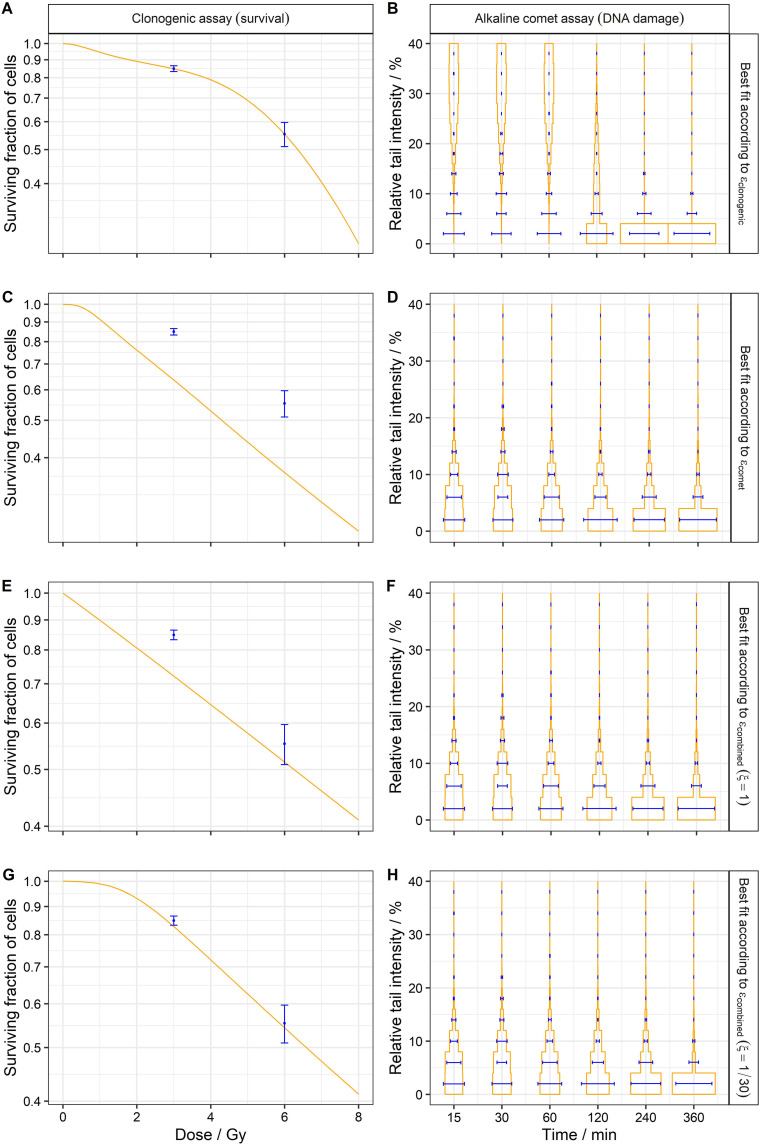
Cell survival (A, C, E, G) and comet distributions (B, D, F, H) simulated results (orange) compared with experimental values (blue) for the Abrams cell line. These results prove that the MHR model (with five parameters) can properly explain the experimental data (62 values). (A, B) Simulations performed for the parameter set with the smallest $\varepsilon_{clonogenic}$ = 2 × 10^−7^ (calculated by Equation (6)): α = 1.37 Gy^−1^, $c_{r}$ = 1.55 h^−1^, $c_{e}$ = 1.06 h^−1^, $\mu_{\Gamma }$ = 5.64 Gy^−1^ and γ = 2.35 h^−1^. (C, D) Simulations performed for the parameter set with the smallest $\varepsilon_{comet}$ = 0.10 (calculated by Equation (5)): α = 0 .17 Gy^−1^, $c_{r}$ = 1.36 h^−1^, $c_{e}$ = 0.31 h^−1^, $\mu_{\Gamma }$ = 5.59 Gy^−1^ and γ = 0.21 h^−1^. (E, F) Simulations performed for the parameter set with the smallest $\varepsilon_{combined}$ ( ξ = 1) = 0.11 (calculated by Equation (7)): α = 0 .17 Gy^−1^, $c_{r}$ = 0.13 h^−1^, $c_{e}$ = 0.19 h^−1^, $\mu_{\Gamma }$ = 0.68 Gy^−1^ and γ = 5.07 h^−1^. (G, H) Simulations performed for the parameter set with the smallest $\varepsilon_{combined}$ ( ξ = 1/30) = 6 × 10^−3^ (calculated by Equation (7)): α = 0 .17 Gy^−1^, $c_{r}$ = 2.25 h^−1^, $c_{e}$ = 0.23 h^−1^, $\mu_{\Gamma }$ = 2.01 Gy^−1^ and γ = 0.25 h^−1^.

The experimental clonogenic and comet data were qualitatively compared with the model results obtained for each objective function (Figure 4). Firstly, the fitting of only clonogenic data resulted in a clear mismatch between the simulated and experimental comet distributions (Figure 4(B)) because the high α value (1.37 Gy^−1^) produced an accumulation of damage in most of the cells (this point will be further discussed later). Additionally, even if the fitting covered both survival points, the survival curve showed a shape that did not correspond with the expected LQ behavior (Figure 4(A)). Therefore, the presented case is unrealistic and must be filtered out by the theoretical discriminators. Secondly, when only comet data were fitted, the model did not cover the experimental survival point at 3 Gy, and the predicted survival curve did not follow an LQ shape as expected (Figure 4(C)). Finally, two different combined fittings were performed: The first approach just added the clonogenic and comet error functions ( ξ = 1). In this case, due to the high number of comet data points compared to the survival ones (60 vs 2), the results are similar to the ones obtained by the comet fitting (Figure 4(E) and (F)). The second approach reduced the contribution of the comet data ( ξ = 1/30). As expected, following this last combined calibration approach, the model could properly describe both clonogenic and comet experimental data (Figure 4(G) and (H)). Furthermore, the fact that the MHR model can adequately fit all the experimental data points with only five model parameters implies that the MHR model can properly cover assays at different biological scales (survival and DNA repair), and, therefore, this work’s relevance goes beyond a simple fitting procedure.

Apart from the qualitative graphical comparison presented in Figure 4, a quantitative analysis of the errors obtained by diverse calibration approaches was performed (Tables 3 and 4). It was observed that the combined fitting sometimes resulted in a better fitting to the experimental data than when separate fittings were performed. Furthermore, it ensured that the calibration results did not deviate in excess from the results obtained by both assays, which did not always happen when the fitting was done separately (Figure 4).

**Table 3.** Errors obtained in comparison with the clonogenic assay experimental data of the Abrams cell line. The model was fitted using different objective functions to obtain 5.000 parameter sets per fitting. The error was calculated with Equation (6) per each model parameter set.

Fitted by	Minimum Error	Maximum Error	Mean Error
Clonogenic	2 × 10^−7^	7 × 10^−3^	10^−3^
Comet	9 × 10^−5^	0.21	0.076
Combined ( ξ = 1)	2 × 10^−4^	0.13	0.037
Combined ( ξ = 1/30)	4 × 10^−6^	0.013	3 × 10^−3^

**Table 4.** Errors obtained in comparison with comet assay experimental data of the Abrams cell line. The model was fitted using different objective functions to obtain 5.000 parameter sets per fitting. The error was calculated with Equation (5) per each model parameter set.

Fitted by	Minimum Error	Maximum Error	Mean Error
Clonogenic	0.23	3.38	1.38
Comet	0.10	0.31	0.18
Combined ( ξ = 1)	0.098	0.56	0.14
Combined ( ξ = 1/30)	0.12	0.71	0.38

### Theoretical Discrimination Results

**Figure 5.**
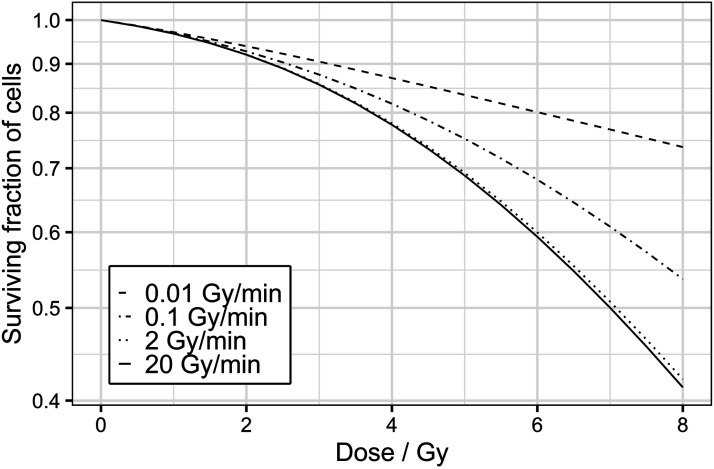
Simulated survival curves for the Abrams cell line at different dose-rates: 0.01 (dashed line), 0.1 (dash-dot line), 2 (dotted line), and 20 Gy/min (solid line). The simulations were performed for a parameter set which passed all the theoretical discriminators: α = 0.26 Gy^−1^, $c_{r}$ = 7.76 h^−1^, $c_{e}$ = 0.83 h^−1^, $\mu_{\Gamma }$ = 0.31 Gy^−1^ and γ = 0.31 h^−1^.

In this section, the capacity of the previously described theoretical discriminators to filter parameter sets was studied. For this purpose, the discriminators were individually and collectively applied to the parameter sets obtained from diverse fittings (Table 5). The results showed that the *additional* and the *survival at different dose-rates* discriminators primarily filtered the parameter sets. Additionally, to a lesser extent, the *low dose-rate* and the *fractionation* discriminators also filtered some sets of parameters. Altogether, when all the theoretical discriminators were applied, only 210 out of 20.000 sets of parameters passed the imposed conditions. Therefore, 99% of the parameter sets were filtered by the dose-rate theoretical discriminators presented in this manuscript. Finally, the theoretical discriminators excluded unrealistic simulated survival curves (see Appendix B in the Supplementary Material). Consequently, a clear dose-rate dependence could be observed in the survival curves simulated with the remaining parameter sets (Figure 5), and the uncertainties in the survival results were greatly reduced (see Appendix C in the Supplementary Material).

**Table 5.** Percentage of parameter sets accepted after applying the different discriminators for the Abrams cell line. The model was fitted using different objective functions to obtain 5.000 parameter sets per fitting filtered by the described theoretical discriminators.

Fitting	Clonogenic	Comet	Combined ( ξ = 1)	Combined ( ξ = 1/30)
Different dose-rates	43.96 %	0.24 %	0.28 %	2.40 %
Low dose-rate	98.14 %	58.36 %	47.48 %	69.20 %
Fractionation	98.58 %	62.94 %	51.82 %	59.50 %
Additional	4.06 %	0.02 %	0.06 %	0.76 %
All	3.86 %	0 %	0 %	0.34 %

### Model Parameters Probability Distributions

**Figure 6.**
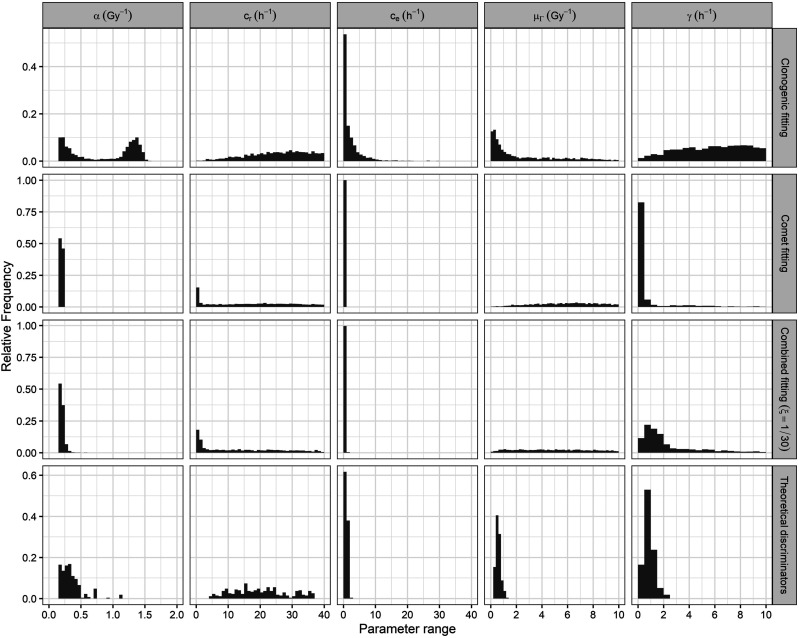
Probability distributions of the MHR parameters obtained for the Abrams cell line by the clonogenic (first row), the comet (second row), and the combined fitting with a weighted factor ξ = 1/30 (third row) and after applying the theoretical dose-rate discriminators (last row).

Due to the high proportion of theoretically discarded parameter sets (Table 5), additional model calibrations (all of them using Equation (7) with ξ = 1/30 as objective function) were needed to increase the statistics of the final parameter probability distributions (the last row of Figure 6). Compared with the results obtained from the clonogenic, comet, and combined calibrations (first, second, and third row of Figure 6, respectively), the ambiguity of $\mu_{\Gamma }$ and γ parameters was strongly reduced. The final probability distribution of the $c_{r}$ parameter reduced its range but still showed a widespread. Similarly, the α probability distribution, despite having a more peaked shape, remained broad. Hence, further research is required to estimate properly α and $c_{r}$ values.

### Model and Theoretical Discriminators Validation

The presented experimental data for the Abrams cell line included time-resolved comet data to get input on the cell repair dynamics. Still the survival curve had only the control and two data points. Given the expected fluctuations of the data, this was not enough to retrieve reliable information about the course of the survival curve (eg, LQ parameters). Therefore, to substantiate the model capabilities and the reliability of some of the theoretical discriminators, dose-rate-dependent measurements for the SiHa cell line were used in this manuscript.

**Figure 7.**
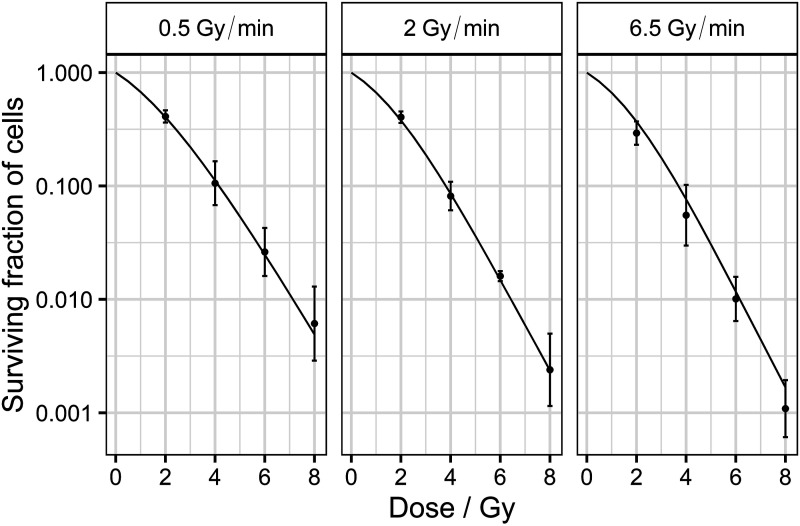
Simulated survival curves (lines) compared with the experimental values (error bars) for the SiHa cell line at different dose-rates: 0.5 (left), 2 (middle), and 6.5 Gy/min (right). The simulations were performed for a parameter set that passed all the theoretical discriminators: α = 0.88 Gy^−1^, $c_{r}$ = 37.5 h^−1^, $c_{e}$ = 21.0 h^−1^, $\mu_{\Gamma }$ = 0.46 Gy^−1^ and γ = 2.10 h^−1^.

Figure 7 demonstrates the capability of the MHR model to cover cell survival at diverse doses and dose-rates. Furthermore, these experimental data allowed us to validate the *survival at different dose-rate* discriminator because they showed a reduction in the survival fraction when the dose-rate was increased (conditions I and II in Table 2) and the difference in the logarithms of the survival between two survival curves at different dose-rates increased with the deposited dose (conditions III and IV in Table 2). Additionally, all the survival curves followed an LQ shape in the logarithmic scale as imposed by condition VIII of Table 2. Finally, the employed dose-rates were not low enough to obtain an exponential survival curve. Nonetheless, the quadratic term obtained from the LQ fitting was 0.028, 0.038, and 0.052 Gy^−2^ for the survival curve at 0.5, 2, and 6.5 Gy/min, respectively. Thus, the survival curve shallowed when the dose-rate decreased, and if the dose-rate is sufficiently reduced, it could be expected to obtain an exponential survival curve as imposed in the *low dose-rate limit* discriminator (condition V in Table 2).

Table 6 exhibits that most of the results obtained from the SiHa calibrations passed the *survival at different dose-rates*, the *low dose-rate* and the *fractionation* discriminators. However, the *additional* discriminator (specifically, condition VII in Table 2) filtered most of the parameter sets. For the calibrations using only one dose-rate, the obtained results were expected not to cover the other two dose-rate survival curves and, hence, would be discarded by the *additional* discriminator. In the case of the calibration considering the three dose-rates, the percentage of parameter sets that passed the *additional* discriminator increased because the results from this calibration covered the experimental survivals better. However, this percentage was still low because the higher number of experimental data complicates the model calibration and increases the probability that one experimental point will not be adequately covered by the model (ie, condition VII in Table 2 will not be fulfilled so that the parameter set will be discarded).

**Table 6.** Percentage of parameter sets accepted after applying the different discriminators. The model was fitted using the SiHa survival curves at different dose-rates to obtain 5.000 parameter sets per fitting filtered by the described theoretical discriminators.

Fitting	0.5 Gy/min	2 Gy/min	6.5 Gy/min	Three Dose-Rates
Different dose-rates	76.98 %	77.86 %	68.50 %	99.86 %
Low dose-rate	99.34 %	99.22 %	99.50 %	99.48 %
Fractionation	97.70 %	94.58 %	91.68 %	100 %
Additional	0 %	0.46 %	0 %	2.74 %
All	0 %	0.44 %	0 %	2.72 %

**Figure 8.**
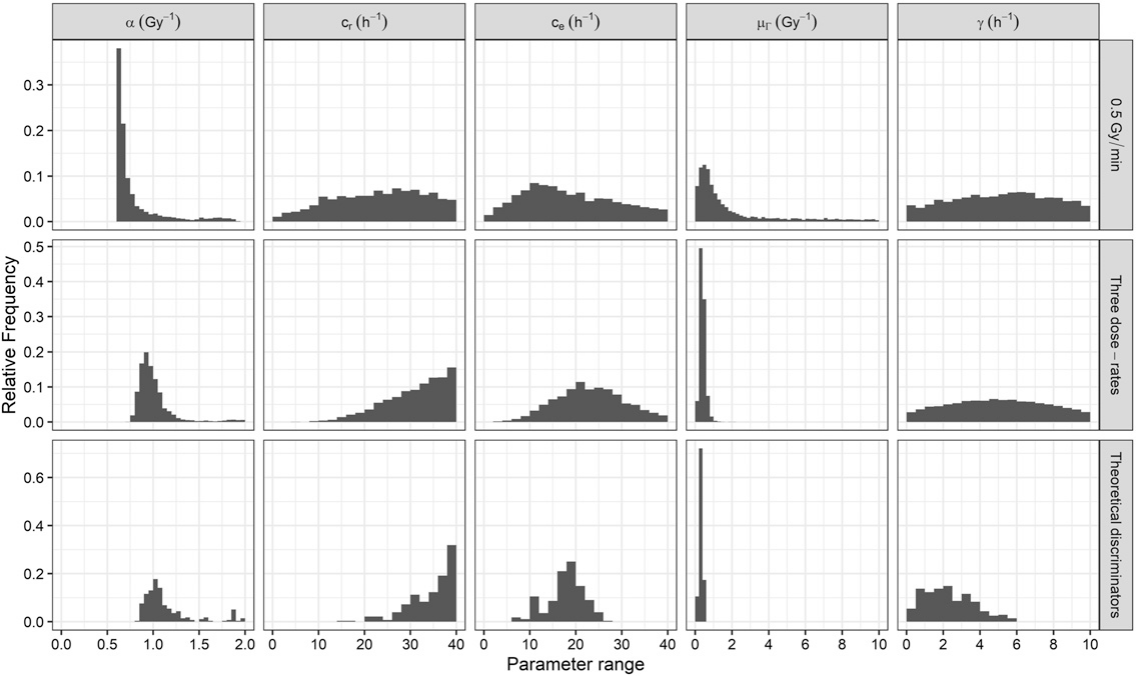
Probability distributions of the MHR parameters obtained for the SiHa cell line by fitting the survival curve at 0.5 Gy/min (upper row), fitting the survival curves at the three dose-rates (middle row), and after applying the theoretical dose-rate discriminators (lower row).

Finally, the SiHa cell line exhibited broader probability distributions (Figure 8) compared to the Abrams cell line (Figure 6). This was probably due to the absence of comet experimental data for the SiHa cell line. Comet experimental data provide dynamic information about cell repair processes, thereby reducing ambiguities in model calibration. This is evident in the peaked probability distributions obtained for the parameters α , $c_{e}$ *,* and γ in the comet and combined calibrations of the Abrams cell line (second and third rows of Figure 6, respectively). Nonetheless, the results indicate that the theoretical discriminators effectively filtered most of the parameter sets (Table 6) and reduced the ranges of the obtained parameters (Figure 8) and the uncertainties in the obtained survival results (see Appendix C in the Supplementary Material). Therefore, although its filtering power is reduced, the proposed methodology remains useful when dose-rate experimental data are included in the model calibration.

## Discussion

In the past, methods such as dose-rate/fractionation experiments were used to obtain an “indirect” insight into cell damage and repair kinetics, and they were proven to be a good alternative when limited accessibility to the relevant dynamic parameters prevents a “direct” measurement *in-vitro* or *in-vivo*. Additionally, this work demonstrates that there are dose-rate effects qualitatively common to most cell lines, and they can be used theoretically to discard unrealistic model dynamics, becoming a helpful tool when experimental data for a specific cell line are missing.

In this manuscript, the canine osteosarcoma Abrams cell line experimental data presented by Weyland et al.^43^ were used as an example to study the effectiveness of the presented theoretical discriminators and to show the ability of the MHR model to fit survival and comet data simultaneously. The theoretical discriminators implemented in this paper discarded 99% of the parameter sets obtained for the MHR model. And, even if ambiguity is still present in the α and $c_{r}$ parameter fittings (ie, more information about the repair kinetics is required), an improved fitting of all model parameters (especially the TBDE repair rate constant γ and the TBDE weighting factor $\mu_{\Gamma }$ ) were obtained (Figure 6). Additionally, the SiHa cervical cancer cell line was used to demonstrate the survival behavior at diverse dose-rates, the capability of the MHR model to cover these data, and the validity of some of the proposed theoretical discriminators. Both calibration results are, in principle, not comparable because Abrams cells (radioresistant canine osteosarcoma) correspond to a completely different cell line than SiHa cells (human cervical squamous cell carcinoma) and, therefore, it can be expected that the model calibrations of both cell lines will differ. However, after including dose-rate dependent theoretical discriminators, a similar result is obtained for the TBDE weighting factor $\mu_{\Gamma }$ in both cell lines may indicate a more general aspect of cellular repair. However, proper testing on several cell lines should confirm this statement.

Therefore, the methodology presented in this manuscript can support understanding the dynamics that govern cell damage and its repair after radiation, especially when the available experimental data is limited. However, a minimum amount of data should be included in the fitting to obtain a decent model calibration. In this regard, the cell damage (eg, comet assay) at multiple time points after irradiation and the survival, including high dose measurements, should be measured to cover the LQL behavior of the survival curve properly.

This study only considered theoretical discriminators based on common qualitative survival dose-rate effects, limiting their filtering effectiveness. However, in future research, the potent classification capabilities of Machine Learning (ML) present an alternative to accelerate the model calibration process and to further discriminate unrealistic model dynamics. Regarding the former, Cevik et al.^51^ demonstrated that an artificial neural network can identify which combinations of model parameters are more likely to generate optimal outputs, thereby decreasing the number of simulation runs needed during calibration and accelerating the overall calibration process. Concerning the latter, an ML algorithm could identify trends and characteristics in survival curves and dose-rate effects that may elude human observation. Yet, adopting ML in this context poses significant challenges. Firstly, unrealistic survival curves must be generated to train the model, and the discrimination criteria may vary depending on how these curves are produced. Secondly, verifying the accuracy of ML’s discrimination criteria can be challenging, requiring rigorous testing across diverse conditions and cell lines to mitigate the risk of over- or under-discrimination in parameter sets. Additionally, ML approaches currently pose more general disadvantages such as extensive training requirements, limited understanding of optimization processes, a lack of clear benchmarks for evaluating solution quality, and uncertainty regarding transfer learning between models. Therefore, while future research in the field may benefit from incorporating ML discrimination methods, the inherent challenges of this methodology should be carefully considered.

Additionally, more information could likely be obtained if the actual experiments were performed and used to fit the MHR model for a certain cell line. Nevertheless, the experimental strategy should be evaluated in advance to obtain the missing biological information. On this matter, the improved model calibration obtained after using the theoretical discriminators can be employed to have a preliminary estimation of the experimental results under diverse conditions and, therefore, to improve the design of future experiments. However, it should be noted that extreme experimental conditions (eg, hypoxia or extremely low and high dose-rates such as FLASH irradiations) should be avoided when applying the presented theoretical discriminators. Although these conditions could be included in the MHR model, they can induce completely different cell damage and repair dynamics, potentially resulting in model calibrations significantly different from those obtained in this study. Finally, the presented theoretical discriminators are only based on cell survival, which is the final treatment outcome, and, therefore, they may miss some dynamic information. Hence, using different dose-rates to perform time-resolve assays like the comet assay may help shed light on this topic.

**Figure 9.**
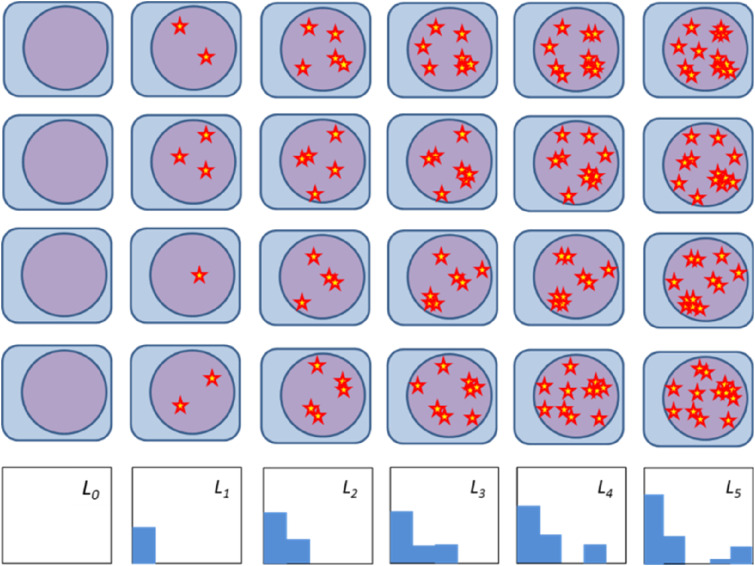
Schematic illustration of the hit distributions in the different populations considered by the MHR model. The population *L*_*0*_ are cells without radiation-induced hits, and in the population *L*_*k*_, cells have at least one cluster with *k* hits (star = hit). Each row corresponds to one cell with a statistically varying number of hits acquired by irradiation with increasing dose (from left to right). The last row shows the histogram for the average number of hits for the depicted four cells.

However, time-resolved data and their interpretation within the MHR model should be carefully considered. Firstly, comparing the hit-population in the MHR model with the DNA fragment amount in the comet tail demonstrates the ability of the MHR model to link assays representing different scales (cell survival and DNA fragment generation and repair). However, the definition of a hit and mapping of the MHR model to specific types of DNA damage is still unclear. Additionally, the chain structure in the MHR model implies that the repair of multiple hits follows a step-by-step process. However, one-by-one repair of isolated/independent hits seems to be an unrealistic biological concept since independent DNA damages are expected to be repaired simultaneously and not one by one (in a step-by-step process). According to Durante et al.^52^ more complex DNA damages have longer repair half times. Therefore, the hits in the MHR model may be interpreted as clusters of hits (Figure 9), which require a more complex repair process that could be represented by a process chain (step-by-step repair). Following this line of argumentation, cells in the population *L*_*k*_ of the MHR model do not have *k* independent hits but at least one (repair-limiting) cluster with *k* hits.

**Figure 10.**
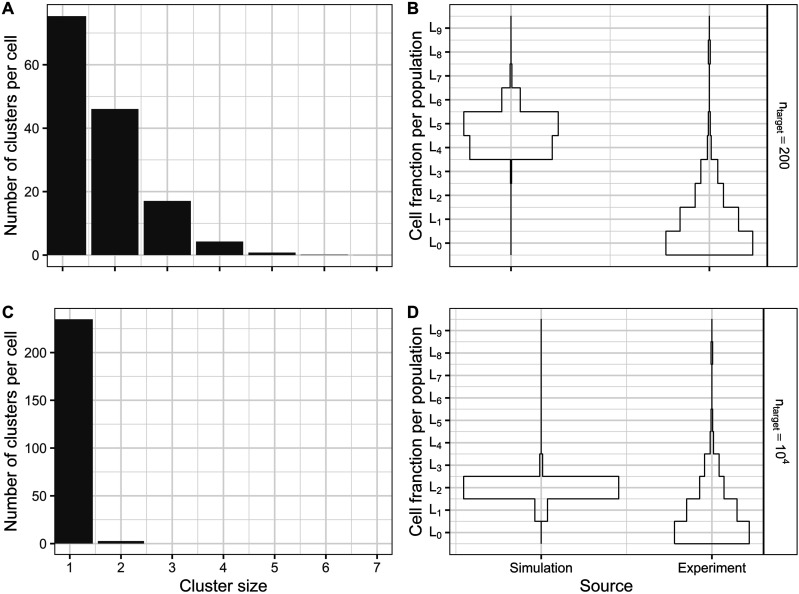
Distributions of clusters with *k* hits in a cell (A, C) and distributions of the number of cells in the different populations *L*_*k*_ (B, D) for a similar dose (6 Gy) as used for comet data fitting by Weyland et al.^43^ The calculations were carried out in steps of 0.5 Gy and applying a linear relationship to the dose. (A, B) Scenario with 200 target sites. The corresponding threshold *q* to achieve approximately 40 hits per Gy for hit induction is 0.9. (C, D) Scenario with 10^4^ target sites. The corresponding threshold *q* to achieve approximately 40 hits per Gy for hit induction is 0.998.

The probability of the appearance of clusters consisting of *k* hits can be calculated easily by a random process, and the resulting histograms of the number of cells with a certain distribution of hits can be compared to time-resolved comet data (Figure 10). Regarding the 3-dimensional structure of the DNA, an interaction/cluster volume (volume wherein 2 or more hits can be considered a cluster) should be defined instead of a range on a linear structure. Vassiliev^53^ estimated the sensitive target volume for photons to a sphere with a 0.1-1 μm radius. This results in a (critical) volume of ca 0.7 μm^3^ for a mean radius of 0.55 μm. Assuming a target site as a spherical interaction volume with a radius of, for example, 94 nm, the target volume can be divided into *n*_*target*_ = 200 such target sites.

On the other hand, assuming a smaller spherical interaction volume with a radius of 25.6 nm, the number of target sites will increase to *n*_*target*_ = 10^4^. In the statistical simulation, the decision of an acquired hit within a target site is based on random numbers in the interval [0,1] and a threshold *q*. If a hit is considered as a double-strand break (DSB), the induction rate is expected to be linear with the dose,^14,51^ which agrees with the linear hit induction rate in the MHR model. Based on the data from Rothkamm et al.^54^ 30-40 DSBs can be expected at 1 Gy. Therefore, the threshold *q* is determined by imposing the condition that 40 hits at a radiation dose of 1 Gy are induced.

Figure 10 shows the histograms for cluster sizes and cell populations at dose levels corresponding to the experimentally observed and fitted data presented by Weyland et al.^43^ For *n*_*target*_ = 200, most of the cells belong to the population *L*_5_ after receiving a dose of 6 Gy (Figure 10(B)). Regarding the distribution of clusters in a cell (Figure 10(A)), only very few (1 or 2) clusters per cell with 5 hits are present. Most clusters consist of 1-2 hits, as it can be expected for a Poisson distribution. However, distributions shifted to higher populations have not been observed by Weyland et al.^43^ Regarding the simulation results with *n*_*target*_ = 10^4^, the appearance of clusters with higher numbers of hits (>2) is unlikely for doses up to 6 Gy (Figure 10(C)). At this dose level, most of the cells (91%) belong to the population *L*_*2*_, and only 7 and 2% of the cells to populations *L*_*1*_ and *L*_*3*_, respectively (Figure 10(D)). Therefore, this case seems more comparable with the comet data of Weyland et al.^43^ since the experimental cell histograms tend more toward base-centered distributions. Nevertheless, the simulated cell population histogram (Figure 10(D)) exhibits a pronounced population inversion, meaning that most cells belong to the population *L*_*2*_. In contrast, in the experimental data and synthetic comets presented by Weyland et al,^43^ only a weak inversion 15-60 min after irradiation or no such inversion is visible.

**Figure 11.**
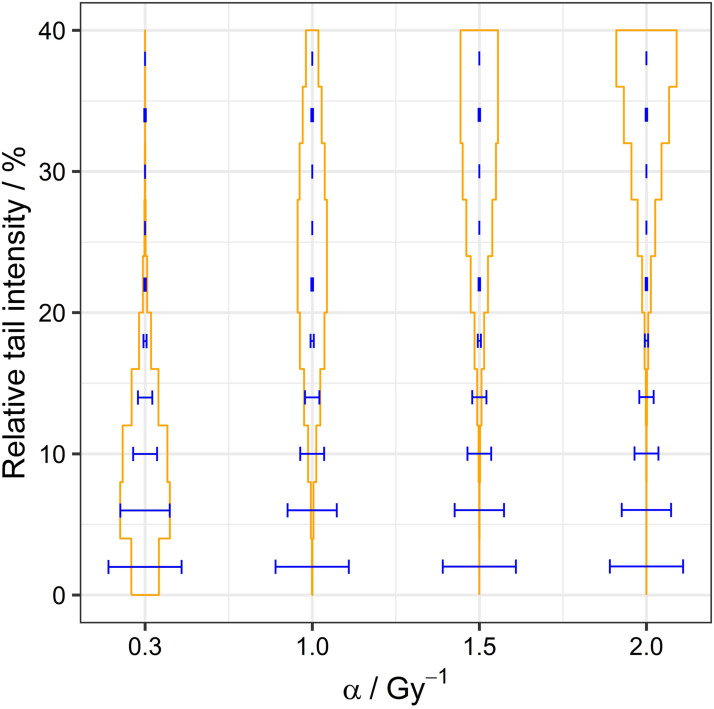
Simulated comet distributions after 6 Gy irradiation for different radiosensitivity α values. The simulated results (orange) are compared with the comet data (blue) fitting performed by Weyland et al.^43^

In conclusion from the presented simulations, clusters with many hits are not probable for many target sites (eg, *n*_*target*_ > 10^4^). Hence, for radiation dose values below 10 Gy, only up to 4 populations (*L*_*0*_-*L*_*3*_) have to be considered in the MHR model. Regarding the reported parameter values found by Scheidegger et al.^1^ and Weyland et al.^43^ for relatively low α values (0.2-0.3 Gy^−1^), 4 populations seem sufficient. On the other hand, high α values of 1-2 Gy^−1^, expected from apoptotic tissues and baseline repair during mitosis, lead to synthetic comets that are not in agreement with observed comets (Figure 11).

As previously mentioned, the population chain is necessary for explaining the experimentally observed survival. Hence, the chain of populations can be limited to 3-4 populations and, at least for higher α values, the hit induction rate has to be adapted for a population *L*_*k*_ (eg, to $-\alpha p^{k}L_{k}$ with a probability *p* for adding hits to an existing cluster) to agree with comet fittings. However, it is important to acknowledge that incorporating the probability p as an additional parameter for model calibration adds an extra degree of freedom to the model, likely increasing the final parameters’ uncertainties. Furthermore, the interpretation of the containers as cell populations with at least one cluster consisting of a defined number of hits is questionable and its suitability must be considered.

In this light, the assignment of comet tail intensity ranges to populations in the MHR model, as proposed by Weyland et al.^43^ should be reviewed. The histograms of cells with a defined amount of DNA fragments in the comet tails presented by Weyland et al.^43^ exhibit more base-centered distributions. However, since an alkaline comet assay was used, fragments generated by SSB may cover the DSB-related distribution. This may also explain the broader distribution in the experimental data compared to the simulated narrow distribution in Figure 10. In addition, the large spread of cells with different amounts of DNA fragments in comet tails observed by Weyland et al.^43^ may be based on different stages in cell cycle and other aspects producing largely variating cellular response: Chromatin condensation may influence the extractability of DNA fragments from the nucleus and fast repair may reduce the amount of fragments shortly after irradiation in a fraction of cells in the analyzed sample.

In general, knowledge about the complex spatiotemporal orchestration of DNA repair may help to find adequate model structures and mappings of these structures to the biological system. Since the cell nucleus is highly structured and functionally compartmentalized, in part due to areas of various degrees of chromatin compaction,^55^ DNA damage repair may be dependent on the localisation in the nucleus, even for a single cell. This may result in a large spread of DNA damage response which motivates the development of simplistic probabilistic models describing the average outcome regarding cell fate. In this framework, the population – chain concept in the MHR model could be considered as such a simplified model but there is still a clear lack of mapping to the biological processes. A similar approach has been proposed by Alemany et al.^56^ for cell differentiation and transcription by using a Fokker-Plank equation for describing the cell fate probability.

## Conclusions

Due to the complexity of the topic, cell repair dynamics cannot be properly studied by only using a single biological assay. Thus, a combination of different *in-vitro* and *in-silico* experiments (including methods that “indirectly” provide dynamic repair information) is probably the best approach to this research. In this context, survival dose-rate dependencies seem to be a good option for obtaining additional dynamic experimental details. Furthermore, these dependencies can be applied as theoretical discrimination criteria to validate dynamic radiobiological models and to improve their fitting. Therefore, dose-rate dependencies in combination with computer simulations are a powerful tool that may also be interesting for other fields beyond the study of DNA repair. Finally, variants of the MHR model (eg, the implementation of clustered hits) should be considered to improve the model interpretation of the available biological data and to allow the model to cover a broader range of experimental conditions.

## Supplemental Material

Supplemental Material - The Use of Survival Dose-Rate Dependencies as Theoretical Discrimination Criteria for *In-Silico* Dynamic Radiobiological Models Supplemental Material for The Use of Survival Dose-Rate Dependencies as Theoretical Discrimination Criteria for *In-Silico* Dynamic Radiobiological Models by Sergio Mingo Barba, Fernando Lobo-Cerna, Przemek M. Krawczyk, Marco Lattuada, Rudolf M. Füchslin, Alke Petri-Fink, and Stephan Scheidegger in Dose-Response
