# Supplementary material for: The Use of Survival Dose-Rate Dependencies as Theoretical Discrimination Criteria for In-Silico Dynamic Radiobiological Models
Source: Dose Response. 2024 Aug 30;22(3):15593258241279906. doi: 10.1177/15593258241279906 (PMC11367615; doi:10.1177/15593258241279906)
Supplement: Supplemental Material - The Use of Survival Dose-Rate Dependencies as Theoretical Discrimination Criteria for In-Silico Dynamic Radiobiological Models [file sj-pdf-1-dos-10.1177_15593258241279906.pdf]

SUPPLEMENTARY MATERIAL

Appendix A: Impact of hit chain cutoff

The chain population of the MHR model must be constrained to a maximum number of hits (k_{max}) and the final simulated results can be influenced by the chosen cutoff. This issue was previously addressed by Scheidegger et al. *(1)*, who found that the effect was negligible for $k_{max} > 4$.

In this work, the same criterion (k_{max}=9) followed by Weyland et al. *(2)* was chosen. To verify its validity, an analysis similar to that conducted by Scheidegger et al. *(1)* was performed. Specifically, the population chain of the MHR model was extended to k_{max} =19, which served as baseline. Then, the survival and the natural logarithm of survival was simulated for various doses (2, 6, 15, and 30 Gy) and dose rates (0.01, 0.1, 2, and 20 Gy/min) using different k_{max} values. These simulations were carried out for all model parameter sets obtained in this study, and the absolute relative error was calculated as follows:

$$\varepsilon_{rel}^{S}(k_{max}) = \left|\frac{S(k_{max}) - S(k_{max} = 19)}{S(k_{max} = 19)}\right| \cdot 100\%$$

$$\varepsilon_{rel}^{logS}(k_{max}) = \left|\frac{logS(k_{max}) - logS(k_{max} = 19)}{logS(k_{max} = 19)}\right| \cdot 100\%$$

Figure S1 illustrates that the most pronounced differences occur at low k_{max} values. As k_{max} increases, the results converge rapidly and extending the population chain does not lead to significant differences. Therefore, it can be concluded that the chosen criterion (k_{max} =9) ensures proper convergence of the simulation results.

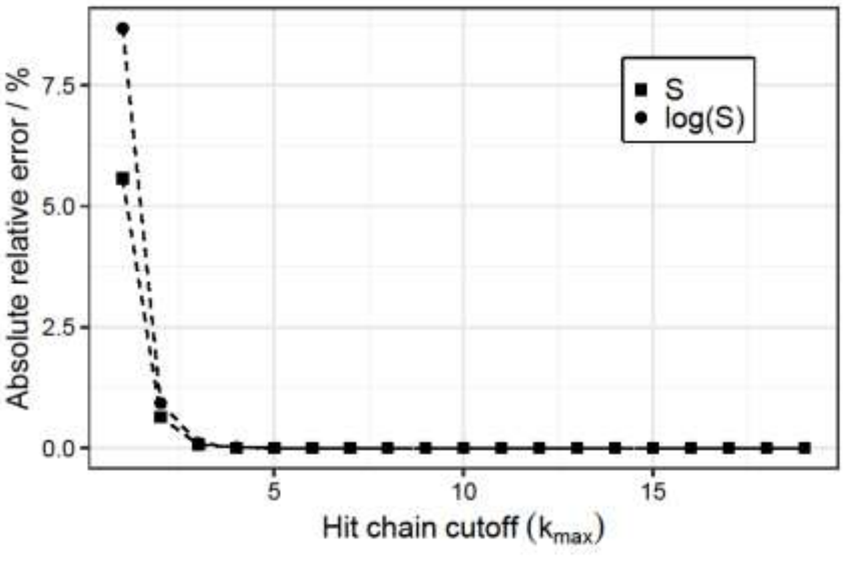

Figure S1: Median absolute relative error for different k_{max} values computing the survival (blue) and the natural logarithm of the survival (orange).

Appendix B: Unrealistic survival curves

In this section, we show several examples of unrealistic survival curves obtained by the different model calibrations when the theoretical discriminators are not applied. Figure S2 illustrates that the MHR model can produce non-LQ curves and opposite dose-rate effects (i.e., the survival increases with the dose-rate), which are not observed in actual survival experiments. The theoretical discriminators presented in this work allow us to filter all these unrealistic scenarios.

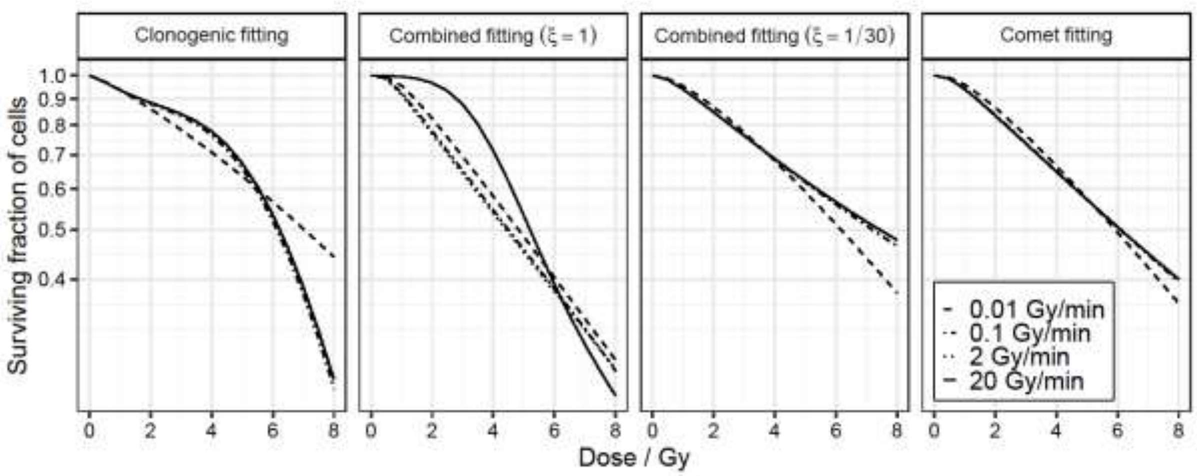

Figure S2: Examples of unrealistic simulated survival curves at different dose-rates: 0.01 (dashed line), 0.1 (dash-dot line), 2 (dotted line), and 20 Gy/min (solid line). Each column corresponds to a certain model calibration.

Appendix C: Uncertainties in the simulated survival

To illustrate how the uncertainties in the parameter distributions affect the simulated survival, Figures 5 and 7 of the manuscript were generated for all the different calibration results presented in this work. Figure S3 presents the mean and the ranges of the obtained survival curves, demonstrating that the theoretical discriminators significantly reduced the variability of the survival curves.

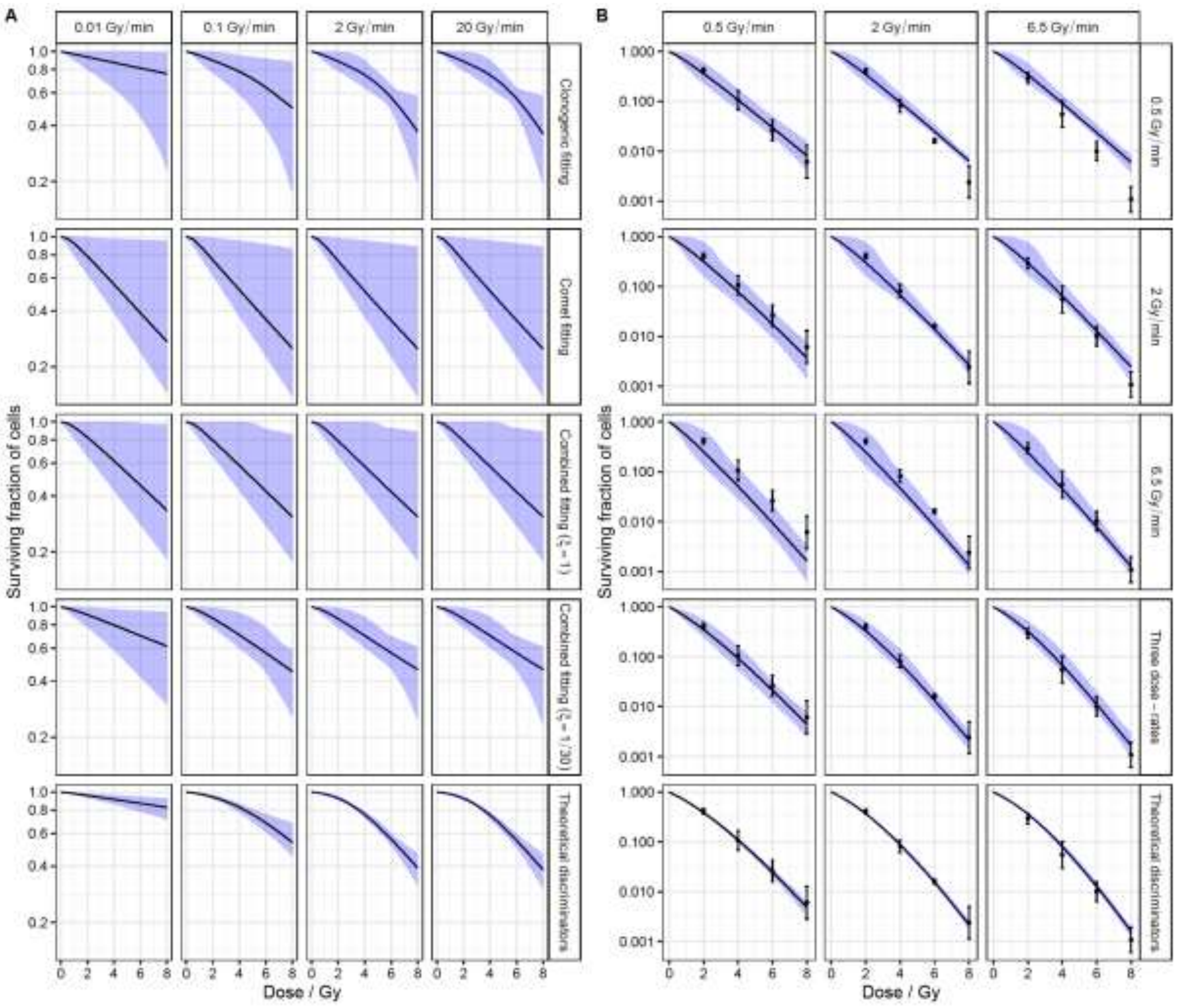

Figure S3: Mean simulated survival (solid lines) and variability (shadowed blue area) obtained for different calibration results. (A) Variability obtained in Figure 5 of the manuscript: Simulated survival curves for the Abrams cell line at different dose-rates (0.01, 0.1, 2, and 20 Gy/min) for different model calibrations and after applying the theoretical discriminators. (B) Variability obtained in Figure 7 of the manuscript: Simulated survival curves compared with the experimental values (error bars) for the SiHa cell line at different dose-rates (0.5, 2, and 6.5 Gy/min) for different model calibrations and after applying the theoretical discriminators.

REFERENCES

1. Scheidegger S, Fuchs HU, Zaugg K, Bodis S, Füchslin RM. Using state variables to model the response of tumour cells to radiation and heat: A novel multi-hit-repair approach. Comput Math Methods Med 2013;2013. https://doi.org/10.1155/2013/587543.

2. Weyland MS, Thumser-Henner P, Nytko KJ, Rohrer Bley C, Ulzega S, Petri-Fink A, et al. Holistic View on Cell Survival and DNA Damage: How Model-Based Data Analysis Supports Exploration of Dynamics in Biological Systems. Comput Math Methods Med 2020;2020. https://doi.org/10.1155/2020/5972594.
